# Evolution of Initial Pharmacologic Treatment of Newly Diagnosed Parkinson's Disease Patients over a Decade in Singapore

**DOI:** 10.1155/2020/6293124

**Published:** 2020-03-30

**Authors:** Shermyn Neo, Sheng Yong Aidan Wong, Hwee Lan Ng, Wei Li, Kay Yaw Tay, Wing Lok Au, Louis Chew Seng Tan

**Affiliations:** ^1^Department of Neurology, National Neuroscience Institute, Singapore; ^2^Department of Research, National Neuroscience Institute, Singapore; ^3^Duke-NUS Medical School, Singapore

## Abstract

**Objective:**

The aim of this study is to compare Parkinson's disease (PD) treatment practices by movement disorder (MD) specialists across a decade, and to determine the factors that influence drug choice for the motor symptoms of PD in newly diagnosed drug-naïve patients.

**Methods:**

This prospective temporal analysis included patients seen at the National Neuroscience Institute in Singapore and diagnosed with PD by MD specialists in the years 2007 and 2017. Primary outcomes were use of specific PD drugs and changes in drug-prescribing patterns. Descriptive analyses and multivariable logistic regression models determined the extent to which patient characteristics were associated with type of PD treatment.

**Results:**

Of 230 patients with PD (mean (SD) age, 66.7 (10.3) years), 131 (57.0%) were male. From 2007 to 2017, the use of ergot dopamine agonists and anticholinergics decreased from 19.3% to 2.0% (*P* < 0.001) and from 12.0% to 2.7% (*P* = 0.004), respectively. The use of monoamine oxidase B inhibitors (MAOBI) increased from 13.3% to 25.2% (*P* = 0.033). The use of levodopa (LD)-sparing strategies decreased nonsignificantly from 33.7% to 24.5% (*P* = 0.133). Overall, 196 (85.2%) patients were initiated on symptomatic monotherapy, with LD being the most commonly prescribed. MAOBI was the most common drug used in combination therapy. Age ≤70 (adjusted OR, 11.9; 95% CI, 4.5–31.5) and Hoehn and Yahr (HY) stage <2 (adjusted OR, 3.4; 95% CI, 1.5–7.7) were independent factors for LD-sparing strategies. Non-LD prescriptions (13 of 92; 14.1%) were more likely to be discontinued compared to LD ones (6 of 149; 4.0%) (*P* = 0.005).

**Conclusions:**

Drug-prescribing patterns in PD have changed significantly through the last decade, influenced by emerging evidence and reports of adverse drug effects. Choosing drugs based on the patient's age and disease severity remain sound guiding principles across the years. It is important that international and national guidelines for pharmacotherapy in PD be updated consistently throughout different socioeconomic settings to optimize care.

## 1. Introduction

Treatment of Parkinson's disease (PD), the second most common neurodegenerative disorder worldwide [1], is largely symptomatic, with its most effective oral therapeutic agent still being levodopa (LD), despite being more than half a century old. The optimal choice of initial symptomatic treatment, however, is unclear, with scientific literature and pivotal clinical trials swinging expert opinion at various turns and igniting fierce debate between LD proponents and those who prefer a LD-sparing strategy [2, 3]. Drug-prescribing patterns may be guided by these evolving scientific evidences.

Few data on temporal trends in Parkinson drug-prescribing patterns in drug-naïve PD patients exist. Most available studies on drug-prescribing patterns in PD have been cross-sectional analyses or use the drug tracer methodology, making it difficult to identify changing patterns in PD drug utilization or patient factors that influence the choice of drug [4–6]. In studies with longitudinal data, analyses were on patients with a varied duration of illness; thus, were not designed to answer which dopaminergic replacement strategy was preferred by physicians as initial therapy [7, 8].

The aim of this study is to compare PD treatment practices by movement disorder (MD) specialists across a decade and to determine the factors that influence drug choice in newly diagnosed drug-naïve PD patients in Singapore. A 10-year comparison was felt to be appropriate as many important pragmatic real-world studies had emerged in the intervening decade [9, 10] with potential impact on drug-prescribing practices.

## 2. Materials and Methods

This study was approved by the Centralized Institution Review Board of the Singapore Health Services.

### 2.1. Data Source

A Movement Disorders database with prospectively collected information has been in existence since 2002 at the National Neuroscience Institute in Singapore (NNI). Patients seen at NNI and diagnosed by MD specialists according to the National Institute of Neurological Disorders and Stroke (NINDS) diagnostic criteria for PD [11] in the years 2007 and 2017 were evaluated. The year 2007 was selected as the dopamine agonists, ropinirole, and pramipexole were only available in the local hospitals 2 years prior. Patients had to be (i) seen by a MD specialist within 1 year of diagnosis, (ii) drug-naïve, and (iii) started on medications within the first 2 clinic visits.

### 2.2. Data Collection

Demographic and clinical information such as age, sex, date of diagnosis, modified Hoehn and Yahr (HY) stage and presence of rest tremor prior to commencement of PD drug, and initial PD drug prescriptions were recorded. For the purpose of this study, any discontinuation of medication(s) within the timeframe of 2 subsequent follow-up clinic visits that occurred within 2 years of the first visit was assumed to be due to medication intolerance. While reasons for discontinuation of treatment were not available for individual patients, it was presumed that medication side-effects usually occur within the first few weeks to months of initiating therapy and is a common reason for discontinuation of therapy (compared to inadequate therapy, in case of which physicians would usually increase the dosage of medication or add on another medication). To ensure that medications were not discontinued because patients defaulted on follow-up visits, their subsequent 2 clinic visits had to occur within 2 years of the index visit. Patients diagnosed in 2017 who had only 1 follow-up visit at the point of study data collection were excluded from this portion of analysis.

From 2007 to 2017, the available oral DA at our institution was bromocriptine, as well as immediate release and sustained release forms of both ropinirole and pramipexole. Available LD formulations included Madopar, Madopar HBS, Sinemet, Sinemet CR, and Credanil. Up to 2017, the only available monoamine oxidase B inhibitor (MAOBI) in the country was selegiline. Total levodopa equivalent dose (LED) per day was calculated for each patient, using standardized conversion formulae.

### 2.3. Statistical Analysis

Statistical analysis was performed using SPSS v21. Descriptive statistics summarized all variables overall, by the year of diagnosis and by the treatment type. One-way ANOVA was used to assess associations between continuous variables of interest and use of a LD-sparing strategy, while Chi-square and Fisher's exact tests were used to assess similar associations with categorical variables. A multiple logistic regression model, with age, sex, HY stage, duration of illness, and presence of rest tremor as covariates, was used to determine independent variables favouring LD-sparing strategies. Linear regression analysis, using the same covariates, was performed to assess factors affecting total daily LED. All statistical tests assumed a 2-sided type one error rate of 0.05.

## 3. Results

### 3.1. Baseline Characteristics

A total of 230 patients met eligibility criteria and were included in the study.  Table 1 summarizes the baseline demographic and clinical characteristics of the study population, cohorted by the year of diagnosis. The mean (SD) age of our patients was 66.7 (10.3) years, and 131 (57.0%) were male. The mean (SD) HY stage prior to commencement of PD drug was 2.3 (0.7). Patients' age, sex, HY stage, duration of illness, and presence of rest tremor at baseline did not differ in the 2007 and 2017 cohorts.

### 3.2. Treatment Patterns in 2007 and 2017

**Figure 1:**
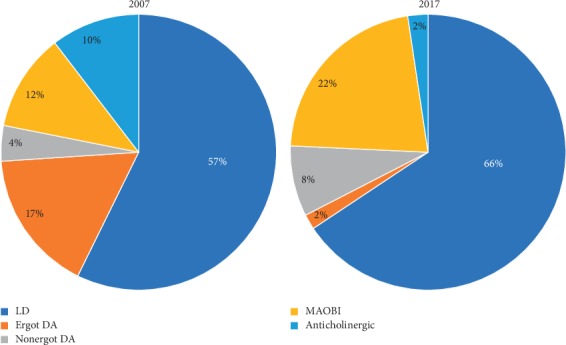
PD drug treatment by year.

As shown in Table 1 and Figure 1, the use of ergot DA and anticholinergics decreased from 19.3% to 2.0% (*P* < 0.001) and from 12.0% to 2.7% (*P*=0.004), respectively, in 2017, compared to 2007. The use of MAOBI, on the other hand, increased from 13.3% in 2007 to 25.2% in 2017 (*P*=0.033). The use of LD-sparing strategies decreased nonsignificantly from 33.7% in 2007 to 24.5% in 2017 (*P*=0.133). The mean daily LED was similar in 2007 and 2017 (198 mg/day vs. 207 mg/day; *P*=0.795).

### 3.3. Drug Combinations

Across both 2007 and 2017 cohorts, 196 (85.2%) patients were initiated on symptomatic monotherapy, with LD being the most commonly prescribed monotherapy (*n* = 151; 77.0%).  Table 2 shows the different drug combinations of patients on dual or triple therapy. MAOBI were most likely PD drugs to be used in combination therapies, with 26 of 34 patients on them.

### 3.4. Factors Affecting Use of LD vs. LD-Sparing Strategies

Compared to LD users, patients on LD-sparing strategies were younger (*P* < 0.001) and had milder disease (*P* < 0.001) (see Table 3). In a multiple logistic regression model, age ≤70 (adjusted OR, 11.9; 95% CI, 4.5–31.5) and HY stage <2 (adjusted OR, 3.4; 95% CI, 1.5–7.7) were independent factors for LD-sparing strategies, after adjusting for sex, duration of illness, and presence of rest tremor. The associations remained significant, after sensitivity analysis by the year of diagnosis.

### 3.5. Factors Affecting Total Daily LED

Table 4 shows the effect of age, sex, HY stage, duration of illness, and presence of rest tremor on LED in the combined cohort. Linear regression analysis demonstrated that every increase of 1 HY stage was associated with a corresponding increase of 52 units in LED (*P* < 0.001).

### 3.6. Discontinuation of Treatment

Non-LD prescriptions (13 of 92; 14.1%) were more likely to be discontinued compared to LD ones (6 of 149; 4.0%) (*P*=0.005). Eight of the discontinued therapies in the non-LD group were MAOBI, while DA accounted for the remaining 5.

## 4. Discussion

In this prospective temporal analysis of drug-naïve PD patients who initiated PD medication, it was observed that the use of ergot DA and anticholinergics had declined in the decade from 2007 to 2017. This was accompanied by a rise in the use of MAOBI, specifically selegiline.

The ergot DA have largely fallen out of use in view of reports of rare but devastating complications of fibrosis, in particular of cardiac valves. While cabergoline and pergolide are associated with the highest risks of fibrotic cardiac valve reactions [12], the same warning to limit dosage of DA and monitor patients for signs of fibrosis while on treatment has been extended to all ergot-derived DA by the European Medicines Agency in 2008.

Anticholinergics with their poor side-effect profile has never been a drug of preference in PD patients, who tend to be older. Greater awareness that anticholinergic use in PD patients is associated with increased risk of adverse health outcomes including fractures, delirium, and hospitalization [13] is likely to have contributed to its decreased usage. In addition, it is increasingly recognized that PD patients may have cognitive impairment even early in their disease course [14]. A recent study showed that inappropriate prescribing patterns are common in older PD patients who may be on anticholinergics and dementia treatment at the same time [15].

MAOBI use in PD rose to prominence at the turn of the millennium, with selegiline postulated to have a possible neuroprotective effect based on its capacity to inhibit MAO-B oxidation of MPTP to MPP + which is toxic to dopaminergic neurons [16]. Furthermore, by blocking free radical formation from the oxidation of dopamine, it is believed that selegiline can slow down neuronal degeneration in PD [17]. Finally, its metabolite, desmethylselegiline, is thought to have antiapoptotic properties [18]. Evidence of its neuroprotective effects in in vitro and in vivo laboratory models led to expert panels recommending the use of selegiline in early PD [19]. Unfortunately, the DATATOP [20] and SINDEPAR [21] trials undertaken to demonstrate this disease-modifying effect have been hampered by confounding symptomatic effects of the antiparkinsonian medications which can have long duration responses that persist for weeks after withdrawal. Nonetheless, the prevailing expert opinion might have led to increasing selegiline prescriptions at our center. This is supported by the finding that the greatest increase in usage of MAOBI was in patients who had also initiated LD. As LD has a far superior symptomatic effect, it may be postulated that the MAOBI was prescribed by our MD specialists for its putative neuroprotective effect. Our center's positive experience with selegiline is reflected in a previous study published in 2011 which found that patients with early PD treated with selegiline for 3 years or more had slower progression of disease as evaluated by HY transition times [22]. Increased utilization of MAOBI was also observed in a recent study across 23 expert care centers [7], with the authors citing good safety profile and ease of use as possible reasons. Rasagiline at a dose of 1 mg per day was showed in an elegantly designed delayed-start trial to have possible disease-modifying effect, but treatment with the 2 mg dose failed to replicate the same result [23]; thus, the U.S. Food and Drug Administration did not label it as such. Since the end of 2017, rasagiline has also been made available at our center. With a convenient once a day dosing, it is anticipated that MAOBI use in Singapore may continue to rise.

Interestingly, the increase in nonergot DA use at our center was not significant. There may be several reasons for this, with results of landmark studies being one. Both pramipexole and ropinirole-treated PD patients developed fewer motor complications then LD-treated ones [24, 25]. At the same time, there were increasing concerns of LD toxicity to the human substantia nigra via the mechanism of auto-oxidation and the generation of free radicals [26]. It was in this climate that national guidelines started to favor DA as initial symptomatic treatment [19, 27]. The growing enthusiasm for DA was, however, dampened by reports of adverse drug effects in the ensuing decade. A case series documented 8 cases of PD patients on pramipexole or ropinirole falling asleep while driving [28]. This was followed by reports of ICD, which were confirmed by a large cross-sectional study of 3090 PD patients demonstrating that DA treatment was associated with a 2-3.5-fold increase in odds of having ICD [10]. Finally, PD-MED was an important pragmatic trial which showed that the long-term risk of developing dyskinesias in LD and LD-sparing groups were similar (36% vs 33%) with no difference in the occurrence of motor fluctuations between groups [9]. LD-treated patients in PD-MED enjoyed persistent benefits in mobility. A case for less emphasis on LD-induced dyskinesia has also been made by Chaudhuri et al. who noted that the prevalence of troublesome dyskinesia requiring treatment is low, with the use of deep brain stimulation and continuous drug-delivery strategies by transdermal or dopaminergic infusion therapies serving to reduce this further [29]. Thus, the subsequent negative literature (and conversely positive literature on LD) may account for the nonsignificant increase in nonergot DA use at our center.

In addition, external factors like cost could have also driven physician prescribing practices. LD remains the cheapest and most heavily government-subsidised PD drug in Singapore. Although ropinirole was subsequently subsided up to 50% in the early 2010 after losing its patent, it remains significantly more expensive than LD, costing 10 times the price. Pramipexole is not subsidised in Singapore. Conversely, while selegiline is also not subsidised, it is considerably cheaper than the nonergot DA and approximates the cost of LD, hence likely facilitating the rise in its usage. The continued use of the ergot DA, bromocriptine, despite its negative side-effect profile may also be explained by its low subsided cost. Although our study was not designed to study external factors influencing prescribing patterns, it is clear that scientific evidence alone does not drive behaviour in clinical practice. In a survey of neurologists on their perceived factors influencing choice of PD medication in Singapore, Tan et al. identified cost as an overriding factor in usage patterns [30]. The aforementioned study by Dubaz et al. also demonstrated no change in DA use between 2010 and 2017, although they did not make a distinction between ergot and nonergot DAs [7].

Daily LED did not differ in 2007 and 2017. This was not surprising as patients in both cohorts were similar in demographics and clinical profile. LED was influenced strongly by disease stage. Interestingly, sex did not affect LED, and both male and female patients received similar mean LED (192 ± 117 mg/day vs. 219 ± 105 mg/day; *P*=0.064). Sex differences in treatment in PD are worth exploring, as several studies have reported that women are more likely to develop dyskinesias, with a lower body weight proposed as the cause [31]. If women and men are receiving similar doses, it may account for higher rates of this motor complication in women.

In our study, age and disease severity were found to be independent clinical factors that influenced whether a patient was initiated on LD or LD-sparing strategies, with older and more severely affected patients more likely to be on LD. This was consistent throughout the decade, supporting the observation that certain basic principles guided PD management by MD specialist at our center. While the debate on LD vs. LD-sparing strategies may not yet be resolved, considering these patient factors remain sound treatment principles. The superior side-effect profile and fewer concerns on LD-related motor complications [32] make LD the preferred choice in the elderly. Like previous studies, we demonstrated that non-LD treatment was not as well-tolerated as LD. In PD-MED, 28% of DA-treated patients and 23% of MAOBI-treated patients discontinued allocated treatment due to side-effects, compared with 2% of LD-treated patients [9].

Our study has several limitations. As this is a single expert care center study in Singapore, the trends observed may not be generalizable to other settings. Our study did not collect nonmotor clinical factors, such as the presence of cognitive impairment or orthostatic hypotension, that are likely to influence to the physician's drug choice. This is because nonmotor symptoms (NMS) in PD were not widely appreciated until recently and documentation of NMS at baseline, prior to drug commencement, was not consistent in 2007. We were not able to study external factors like cost and government policy that may have important impact on PD drug-prescribing patterns. Finally, reasons for discontinuation of drug therapy were not available for individual patients, although care was taken to ensure that this was not due to patients being lost to follow-up. Nonetheless, this is the first study on temporal changes in prescribing patterns in drug-naïve PD patients and does shed light on how scientific evidence influences clinical practice. Our use of a current and comprehensive dataset that is updated regularly with clinical visit data is another strength.

In conclusion, drug-prescribing patterns in PD have changed significantly through the last decade with a shift away from LD-sparing strategies, possibly influenced by emerging evidence and postmarketing surveillance reports. Key guiding principles of choosing drugs based on the patient's age and disease severity remained consistent across the years. It is important that international and national guidelines for drug-prescribing in PD be updated and consistent throughout different socioeconomic settings to optimize care.

## Figures and Tables

**Table 1:** Patient characteristics and treatment based on the year of diagnosis.

	Entire cohort *n* = 230	2007 cohort *n* = 83	2017 cohort *n* = 147	*P* value
Mean (SD) age, years	66.7 (10.3)	66.4 (10.1)	66.9 (10.5)	0.737

Male, *n* (%)	131 (57.0)	48 (57.8)	83 (56.5)	0.840

Mean (SD) HY stage	2.3 (0.7); *n* = 222	2.4 (0.7); *n* = 76	2.2 (0.6); *n* = 146	0.196

Mean (SD) duration of illness, months	65.5 (57.0); *n* = 228	59.7 (56.4); *n* = 82	68.6 (57.3); *n* = 146	0.246

Rest tremor present	125 (61.3); *n* = 204	44 (69.8); *n* = 63	81 (57.4); *n* = 141	0.093

Monotherapy, *n* (%)	196 (85.2)	70 (84.3)	126 (85.7)	0.778

Treatment, *n* (%)				

LD				
Total^*∗*^	166 (72.2)	55 (66.3)	111 (75.5)	0.133
Monotherapy	151 (65.7)	53 (63.9)	98 (66.7)	

Ergot DA				
Total	19 (8.3)	16 (19.3)	3 (2.0)	**<.001**
Monotherapy	10 (4.3)	10 (12.0)	0 (0.0)	

Nonergot DA				
Total^*∗*^	18 (7.8)	4 (4.8)	14 (9.5)	0.202
Monotherapy	10 (4.3)	1 (1.2)	9 (6.1)	

MAOBI				
Total^*∗*^	48 (20.9)	11 (13.3)	37 (25.2)	**0.033**
Monotherapy	22 (9.6)	4 (4.8)	18 (12.2)	

Anticholinergic				
Total^*∗*^	14 (6.1)	10 (12.0)	4 (2.7)	**0.004**
Monotherapy	3 (1.3)	2 (2.4)	1 (.07)	

Mean (SD) LED, mg/day	204 (113)	198 (106)	207 (117)	0.566

DA, dopamine agonist; HY, Hoehn and Yahr; LD, levodopa; LED, levodopa equivalent dose; MAOBI, monoamine oxidase type B inhibitor; SD, standard deviation. ^*∗*^Combination and monotherapy.

**Table 2:** Drug combinations for patients on dual/triple therapy in 2007 and 2017.

Drug combinations	*n* = 34
Dual therapy	
LD + DA	2
LD + MAOBI	10
LD + anticholinergic	2
DA + MAOBI	10
DA + anticholinergic	4
MAOBI + anticholinergic	5

Triple therapy	
LD + DA + MAOI	1

DA, dopamine agonist; LD, levodopa; MAOBI, monoamine oxidase type B inhibitor.

**Table 3:** Univariate analysis of factors determining the use of LD vs. LD-sparing strategies.

	LD	LD-sparing	*P* value
Age	70.3 ± 8.8	57.5 ± 8.1	**<.001**
Male, *n* (%)	91 (54.8)	40 (62.5)	0.292
HY stage, mean (SD)	2.4 (0.7)	1.9 (0.5)	**<.001**
Mean duration of illness (SD), months	68.9 (61.2)	56.8 (43.6)	0.148
Rest tremor present, *n* (%)	84 (57.5)	41 (70.7)	0.082

HY, Hoehn and Yahr; LD, levodopa. ^#^All characteristics were analyzed as continuous variables except sex and presence of rest tremor. Results of multivariate analysis including all factors listed in the table are reported in the main text.

**Table 4:** Factors affecting total daily LED.

	Effect estimate	*P* value
Age	1.312	0.085
Male	9.612	0.533
HY stage	51.626	**<.001**
Duration of illness	−3.777	0.806
Presence of rest tremor	−0.67	0.636

HY, Hoehn and Yahr.

## Data Availability

Data will be shared on request from any qualified investigator.
